# Progress Toward Poliomyelitis Eradication — Nigeria, January 2014–July 2015

**DOI:** 10.15585/mmwr.mm6432a5

**Published:** 2015-08-21

**Authors:** Andrew Etsano, Rajni Gunnala, Faisal Shuaib, Eunice Damisa, Pascal Mkanda, Johnson M. Ticha, Richard Banda, Charles Korir, Ana Elena Chevez, Ogu Enemaku, Melissa Corkum, Lora B. Davis, Gatei-wa Nganda, Cara C. Burns, Steven G.F. Wassilak, John F. Vertefeuille

**Affiliations:** 1 National Primary Health Care Development Agency, Federal Republic of Nigeria; 2 Global Immunization Division, Center for Global Health, CDC; 3 Federal Ministry of Health, Federal Republic of Nigeria; 4 World Health Organization, Nigeria Office; 5 United Nations Children’s Fund, Nigeria Office; 6 Division of Viral Diseases, National Center for Immunization and Respiratory Diseases, CDC

Since the 1988 launch of global poliomyelitis eradication efforts, four of the six World Health Organization (WHO) regions have been certified polio-free (1). Nigeria is one of only three countries, along with Afghanistan and Pakistan, where transmission of wild poliovirus (WPV) has never been interrupted. During 2003–2013, northern Nigeria served as a reservoir for WPV reintroduction into 26 previously polio-free countries (2). In 2012, the Nigerian government launched a national polio eradication emergency plan (3) to intensify efforts to interrupt WPV transmission. This report describes polio eradication activities and progress in Nigeria during January 2014–July 2015 and updates previous reports (2–4). No WPV cases have been reported to date in 2015, compared with a total of six cases reported during 2014. Onset of paralysis in the latest reported WPV type 1 (WPV1) case was July 24, 2014. Only one case of circulating vaccine-derived poliovirus type 2 (cVDPV2) has been reported to date in 2015, compared with 20 cVDPV2 cases during the same period in 2014. Pending final laboratory testing of 218 remaining specimens of 16,617 specimens collected since January 2015, Nigeria could be removed from the WHO list of polio-endemic countries in September 2015. Major remaining challenges to the national polio eradication program include sustaining political support and program funding in the absence of active WPV transmission, maintaining high levels of population immunity in hard-to-reach areas, and accessing children in security-compromised areas of the northeastern states.

## Vaccination Activities

Nigeria’s routine immunization program includes vaccination with trivalent (types 1, 2, and 3) oral poliovirus vaccine (tOPV) at birth and ages 6, 10, and 14 weeks. In 2014, WHO and the United Nations Children’s Fund estimated national 3-dose tOPV coverage (tOPV3)* among children aged <12 months to be 66% (5). In February 2015, inactivated polio vaccine (IPV) was introduced into the routine immunization program and is being rolled out in phases that initially prioritized eleven polio high-risk states† (6), and as of July, had been introduced in 35 of Nigeria’s 36 states as well as the Federal Capital Territory. This is part of a global plan to provide immunity to type 2 poliovirus (the most common type of cVDPV) in all OPV-using countries, before a synchronized switch from tOPV to bivalent OPV (bOPV), which contains OPV types 1 and 3 (7).

During January 2014–July 2015, 14 supplemental immunization activities (SIAs)§ were conducted in Nigeria. The majority of the 10 subnational SIAs used bOPV, although some local government areas (LGAs) (equivalent to districts) at increased risk for cVDPV2 emergence used tOPV. Of the four national SIAs conducted during this period, one used tOPV, one used bOPV, and two used bOPV in some states and tOPV in others, depending upon polio risk profiles. During SIAs using both tOPV and IPV in selected high-risk states and LGAs from June 2014 through May 2015, approximately 4.4 million IPV doses were administered in high-risk communities.

**FIGURE 1:**
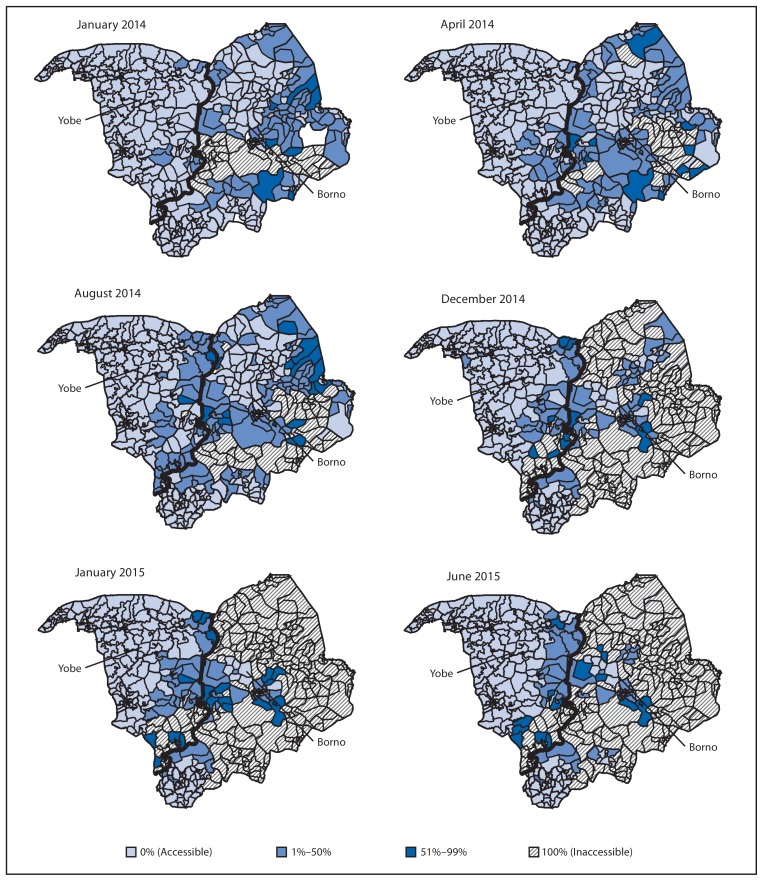
Areas inaccessible to vaccination teams, by proportion of inaccessible settlements — Borno and Yobe states, northern Nigeria, January 2014–June 2015

A number of strategies were implemented during January 2014–July 2015 to enhance the quality of SIAs and to further engage communities, including continued use of an accountability dashboard tool,¶ directly observed polio vaccination,** health camps,†† and social mobilization by volunteer community mobilizers, religious and traditional leaders, and polio survivors, who continue to assist in reducing noncompliance. Although areas of inaccessibility caused by political insurgency increased in places such as Borno, Yobe, and northern Adamawa states (Figure 1), additional innovative strategies continue to be implemented, including permanent health teams made up of women who deliver OPV to households within their communities, transit-point vaccination, vaccination in camps for internally displaced persons, short-interval SIAs that take advantage of intermittent access to normally inaccessible areas, and vaccination of children attending malnutrition treatment centers.

SIA quality is assessed using lot quality assurance sampling (LQAS)§§ surveys to estimate whether OPV coverage in the surveyed area is at or above a threshold of 90%. During January 2014–July 2015, the number of LGAs conducting LQAS surveys in the 11 high-risk states increased from 207 to 226. During the same period, the proportion of LGAs passing at or above the 90% threshold increased from 47% to 75%, the proportion of LGAs at the 80%–89% level decreased from 34% to 22%, and the proportion of LGAs below the 80% level decreased from 18% to 3%.

## Poliovirus Surveillance

### Acute flaccid paralysis surveillance

**FIGURE 2:**
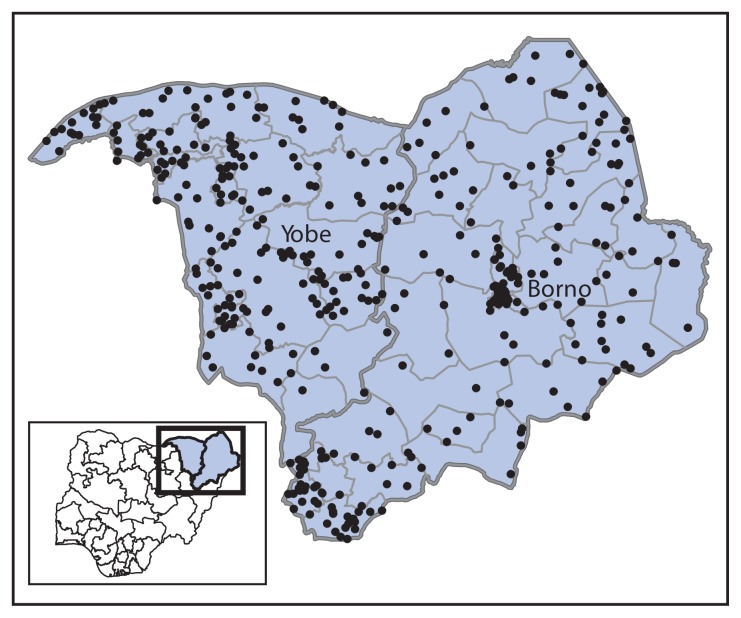
Cases of nonpolio acute flaccid paralysis reported (N = 435)* — Borno and Yobe states, northeast Nigeria, January–July 2015 * Each dot represents one case.

Polio surveillance relies on laboratory-supported acute flaccid paralysis (AFP) case detection and confirmation. Two indicators are used to assess the quality of AFP surveillance: documentation of a nonpolio AFP (NPAFP) rate of two or more cases per 100,000 population aged <15 years (indicating satisfactory sensitivity) and collection of adequate stool specimens from ≥80% of persons with AFP (1). Nigeria’s NPAFP rate for 2014 was 14.8 per 100,000,¶¶ and 97% of AFP cases had adequate stool specimen collection. For 2015, the annualized NPAFP rate was 13 cases per 100,000, and adequate stool specimens were collected for 99% of AFP cases. All 11 high-risk states exceeded both indicator standards in 2014 and continue to do so in 2015. The proportion of reporting LGAs within these states that met both standards was 98% in 2014 and remains 98% to date in 2015. Efforts have been made to enhance surveillance in insecure areas within Borno and Yobe states by adding reporting sites, increasing the number of community informants, and monitoring the performance of surveillance weekly at the national level. As a result, the 2015 NPAFP rate per 100,000 population <15 years was 17.0 for Borno and 27.7 for Yobe (Figure 2).

### Environmental surveillance

AFP surveillance is supplemented by environmental surveillance; samples are taken from effluent sewage sites every 2–4 weeks for poliovirus testing. By July 2015, environmental surveillance was being conducted in 38 sites, mostly in northern Nigeria: Borno (four sites), Kaduna (three), Kano (five), Lagos (five), Sokoto (four), the Federal Capital Territory (two), Kebbi (three), Katsina (three), Jigawa (three), Yobe (three), and Adamawa (three). In 2014, WPV1 was detected in one sewage sample collected in May in Kaduna, and cVDPV2 was detected in 54 sewage samples: 14 from Kano (last detected in July 2014); 13 from Borno (June); 12 from Sokoto (August); 11 from Kaduna (October); two from Katsina (October); and one each from Jigawa and Yobe (November). Borno had no further positive environmental samples after mid-2014, following the introduction of IPV and use of tOPV in SIAs in the state. During January–July 2015, cVDPV2 was identified in one sewage sample collected from Kaduna (March).

## Polio Incidence

### WPV and cVDPV polio cases

**FIGURE 3:**
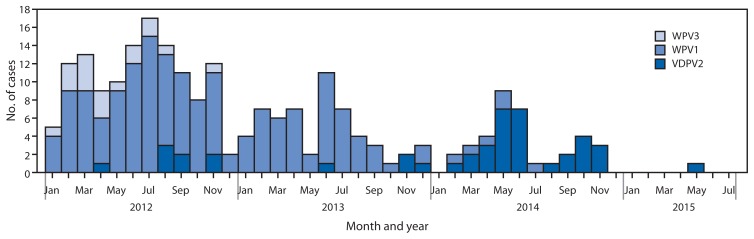
Number of cases of wild poliovirus type 1 (WPV1), wild poliovirus type 3 (WPV3), and vaccine-derived poliovirus type 2 (VDPV2), by month — Nigeria, January 2012–July 2015

No WPV1 cases have been reported in Nigeria to date in 2015. During 2014, six WPV1 cases were reported, 53 were reported during 2013, and 122 were reported during 2012 (Figure 3). The six WPV1 cases in 2014 were geographically limited to five in Kano and one in Yobe state; onset of paralysis in the last reported WPV1 case was July 24, 2014. The last WPV type 3 case was reported in November 2012. One cVDPV2 case has been reported to date in 2015 in the Federal Capital Territory, with a paralysis onset date of May 16. During 2014, 30 cVDPV2 cases were reported, compared with four cases in 2013. Six polio-compatible*** cases have been reported in 2015 thus far, compared with 21 during the same period in 2014. Overall in 2014, 35 compatible cases were reported.

### Genomic sequence analysis

Since 2012, the genetic diversity of WPV in Nigeria has declined. Among eight genetic clusters of poliovirus detected in 2012, four were identified in 2013; among these, two active clusters were found in 2014. Genomic sequence analysis can also be used to identify AFP surveillance gaps not otherwise shown by surveillance performance indicators. In areas with good surveillance, isolates from environmental sampling are usually closely related, having >98.5% nucleotide sequence identity in the coding region of the major capsid protein, VP1. Poliovirus isolates with a nucleotide difference of ≥1.5% in the VP1 coding region indicate undetected chains of transmission. During 2012, 2013, and 2014, VP1 nucleotide differences of ≥1.5% were found in 10 of 103, 10 of 53, and two of six sequenced WPV1 isolates, respectively. During 2014, the proportion of cVDPV2 isolates with a VP1 nucleotide difference of ≥1.5% (7.8%) was similar to that in 2013 (6.8%). The isolate from the single 2015 cVDPV2 case is genetically linked to viruses that were first detected in Kaduna in 2014. For 2015, a VP1 nucleotide difference of ≥1.5% was found in one isolate (of seven sequenced isolates) from an environmental sample taken during March in Kaduna state; it was genetically linked to Nigerian viruses associated with the major cVDPV2 lineage group that first emerged in 2005 (8).

## Discussion

Since establishing a polio emergency operations center and implementing a national emergency polio eradication action plan supported with global partners in 2012, Nigeria has experienced a progressive decrease in WPV1 cases. The success of strategies implemented to improve SIA quality and increase access to hard-to-reach children is reflected in improved LQAS survey data. Despite a decline in genetic diversity of WPV1 during 2012–2014 and achievement of surveillance performance indicators at the national level, virologic data indicated persistent gaps in AFP surveillance quality even in 2014. Nonetheless, allowing for delays in obtaining results from the remaining 218 laboratory specimens, if no WPV is identified in AFP cases or environmental samples, Nigeria stands poised for imminent removal from the WHO list of polio-endemic countries.

For the African region to be certified polio-free, all countries in the region will have to maintain a zero WPV1 case incidence for ≥36 months with high-quality surveillance. Continued strengthening of surveillance is required, including active case finding and close monitoring of polio-compatible cases, which might indicate missed transmission.

Nigeria is at risk for persistent cVDPV2 transmission because of low routine immunization coverage (9) and predominant use of bOPV in SIAs, which could lead to gaps in immunity to type 2 viruses. Efforts to strengthen routine immunization are ongoing in polio high-risk LGAs with existing polio infrastructure; these include building capacity and increasing accountability for routine immunization service provision at the health facility level. Interrupting cVDPV2 transmission will also require increased use of tOPV in SIAs, boosting immunity to type 2 polioviruses with IPV, and strengthening outbreak response to any newly identified VDPV. Five of the next six planned SIAs will use tOPV.

The national polio program will need to continue to manage the challenges posed by the insecurity in areas of northeastern Nigeria where many children remain inaccessible to vaccination services. Innovative strategies, including use of permanent health teams, transit-point vaccination, short interval SIAs, and vaccination of children who access point of care sites, in addition to monthly security risk assessments, will be key to achieving consistent coverage in these areas. Nigeria’s polio program, in collaboration with international partners, will need to continue to advocate for its eradication priorities, to ensure sustained support during the post-transmission period and after changes in national political leadership.

Polio program legacy planning in Nigeria has begun. Documentation of lessons learned during the challenging fight to eradicate polio is critical because this knowledge can shape future approaches to global health (10). This process includes evaluation of current programs, planning for post-certification transition of polio assets and further use of polio eradication infrastructure to strengthen routine immunization and other national public health priorities. Continued partner and government support will be essential for creating the polio eradication legacy in Nigeria, and for maintaining a polio-free African region.

**Summary**

What is already known on this topic?

Nigeria is one of only three countries in the world where wild poliovirus (WPV) transmission has never been interrupted. Nigeria’s 2012 national polio eradication emergency plan has led to improved quality of vaccination efforts, increased accountability, and a decline in WPV cases from 2013 to 2014.

What is added by this report?

The number of WPV cases decreased from six during 2014 to zero through July 2015. Only one reported case of circulating vaccine-derived poliovirus type 2 (cVDPV2) has been reported to date in 2015; however, the number of reported cVDPV2 cases increased from 2013 to 2014. Although genetic diversity declined during 2012–2014 and surveillance performance indicators have been met, gaps in surveillance persist.

What are the implications for public health practice?

Challenges include maintaining political support and program funding in the absence of active WPV transmission, maintaining high levels of population immunity in hard-to-reach areas, and accessing children in security-compromised parts of the northeastern states. Pending the clearance of the 218 remaining laboratory results, Nigeria is poised to be removed from the World Health Organization’s list of polio-endemic countries in September 2015. When this occurs, certification of a polio-free Africa region by the end of 2017 will be achievable. Documenting lessons learned during this fight for polio eradication will allow Nigeria to successfully use existing infrastructure to address other public health problems.

## Footnotes

* Coverage with the third dose of diphtheria-tetanus-pertussis vaccine is used as a surrogate for routine immunization coverage because reported OPV coverage can include doses given during SIAs.

† Polio high-risk states in northern Nigeria: Bauchi, Borno, Jigawa, Kaduna, Kano, Katsina, Kebbi, Niger, Sokoto, Yobe, and Zamfara.

§ Mass campaigns conducted for a few days, during which 1 dose of OPV is administered to all children aged <5 years, regardless of vaccination history. Campaigns can be conducted nationally or subnationally.

¶ Monitors SIA preparations and execution at the LGA level.

** Outside household vaccination in areas with a high proportion of missed children that features entertainers to promote positive vaccination experiences.

†† Community level fixed-point vaccination centers providing various primary health care services during SIAs.

§§ A clustered LQAS methodology is used to assess SIA quality by sampling the target population of children at the LGA level and documenting finger markings indicative of OPV receipt. A sample is drawn from six wards (geopolitical subunits) within the LGA, with 10 children in a single settlement selected at random from each sampled ward. This yields a total sample of 60 children per LGA. LGAs are classified into one of four classifications based on the number of unmarked children found: 0–3 high pass (dark green); 4–8 pass (light green); 9–19 unacceptable (yellow); and >19 fail (red). A detailed description of the methodology is available at http://www.polioeradication.org/Portals/0/Document/Research/OPVDelivery/LQAS.pdf.

¶¶ Calculated using WHO African Region population estimates.

*** A case in which two adequate stool specimens were not collected from an AFP case within 2 weeks of the onset of paralysis, for which a panel of experts considers the clinical presentation to be compatible with polio and 1) an acute paralytic illness is reported with polio-compatible residual paralysis at 60 days; 2) death takes place within 60 days; or 3) the case is lost to follow-up. Case definitions are available at http://www1.paho.org/english/HVP/HVI/hvp_fg_pol.pdf.
